# Subcellular Distribution of S-Nitrosylated H-Ras in Differentiated and Undifferentiated PC12 Cells during Hypoxia 

**DOI:** 10.22074/cellj.2017.4546

**Published:** 2017-08-19

**Authors:** Tamar Barbakadze, Galina Goloshvili, Nana Narmania, Elene Zhuravliova, David Mikeladze

**Affiliations:** 1 Institute of Chemical Biology, School of Natural Sciences and Engineering, Ilia State University, Tbilisi, Georgia; 2 Department of Biochemistry, I. Beritashvili Center of Experimental Biomedicine, Tbilisi, Georgia

**Keywords:** Cell Hypoxia, Nitric Oxide, H-Ras, Mitochondria, ATP

## Abstract

**Objective:**

Hypoxia or exposure to excessive reactive oxygen or nitrogen species could induce S-nitrosylation of various target proteins, including GTPases of the Ras-superfamily. Under hypoxic conditions, the Ras-protein is translocated to the cytosol and interacts with the Golgi complex, endoplasmic reticulum, mitochondria. The mobility/translocation of Ras depend on the cells oxidative status. However, the importance of relocated S-nitrosylated-H-Ras (NO-H-Ras) in proliferation/differentiation processes is not completely understood. We have determined the content of soluble- and membrane-bound-NO-H-Ras in differentiated (D) and undifferentiated (ND) rat pheochromocytoma (PC12) cells under hypoxic and normoxic conditions.

**Materials and Methods:**

In our experimental study, we analyzed NO-H-Ras levels under hypoxic/normoxic conditions in membrane and soluble fractions of ND and D PC12 cells with/without nitric oxide donor, sodium nitroprusside (SNP) treatment. Cells were analyzed by the S-nitrosylated kit, immunoprecipitation, and Western blot. We assessed the action of NO-H-Ras on oxidative metabolism of isolated mitochondria by determining mitochondrial hydrogen peroxide generation via the scopoletin oxidation method and ATP-production as estimated by the luminometric method.

**Results:**

Hypoxia did not influence nitrosylation of soluble H-Ras in ND PC12 cells. Under hypoxic conditions, the nitrosylation of soluble-H-Ras greatly decreased in D PC12 cells. SNP didn’t change the levels of nitrosylation of soluble-H-Ras, in either hypoxic or normoxic conditions. On the other hand, hypoxia, per se, did not affect the nitrosylation of membrane-bound-H-Ras in D and ND PC12 cells. SNP-dependent nitrosylation of membrane-bound-H-Ras greatly increased in D PC12 cells. Both unmodified normal and mutated H-Ras enhanced the mitochondrial synthesis of ATP, whereas the stimulatory effects on ATP synthesis were eliminated after S-nitrosylation of H-Ras.

**Conclusion:**

According to the results, it may be proposed that hypoxia can decrease S-nitrosylation of soluble-H-Ras in D PC12 cells and abolish the inhibitory effect of NO-H-Ras in mitochondrial oxidative metabolism.

## Introduction

Ras proteins are low molecular mass GTPases that regulate cellular functions of proliferation, differentiation, migration, and apoptosis (1). Ras proteins are proto-oncogenes frequently mutated in human cancers. Ras are regulated by a series of post-translational modifications that include farnesylation, methylation, and palmitoylation. These modifications are essential for redistribution of Ras isoforms between the cell surface and endomembrane compartments such as Golgi complex, endoplasmic reticulum, and mitochondria (2). Compartmentalization influences Ras signaling, since Ras isoforms located in the cell surface and endomembranes generate different signal outputs (3). 

The Ras protein contains several cysteine (Cys) residues, which can be nitrosylated by nitric oxide (NO) (4). Among these Cys residues, the most significant are Cys118, located near the guanine nucleotide-binding motif, and Cys181, Cys184, and Cys186, located in the C-terminal region of the protein. S-nitrosylation of Cys in H-Ras depends on the concentration of NO. Low levels of NO (<0.1 mM) can nitrosylate Cys118, whereas high concentrations (>0.1 mM) have the ability to modify terminal Cys (5,7). The terminal Cys are typically targeted for lipid modifications and considered potential sites for regulatory nitrosylation reactions (7). Treatment of cells with the nitrosylating agents decreases palmitoylation of H-Ras (8). An analysis of association/dissociation kinetics of H-Ras with phospholipids has shown that the pretreatment of farnesylated H-Ras protein by S-nitroso- cysteine decreased the incorporation of H-Ras in phospholipids (9). Elevation of oxidants that may occur during oxidative/metabolic stress induces oxidation of thiols in Cys181/184 that could change the palmitoylation status of H-Ras (10) and subsequently its intracellular localization. Taking into account the NO-modifications of Ras in different subcellular compartments that regulate different downstream signaling pathways (6), the S-nitrosylation of H-Ras is critical in Ras-driven pathologies such as cancer. 

Accumulating evidence indicates that dysregulated, diminished or excessive S-nitrosylation may be implicated in a wide range of pathophysiological conditions (11). Severe cellular stress, initiated by hypoxia or exposure to excessive reactive oxygen/ nitrogen species, can induce S-nitrosylation of various proteins and lead to disarrangement of stress signals (12). Hypoxia can stimulate the production of ROS by mitochondria, which in turn, activate hypoxia-inducible transcription factor 1 (HIF1) (13,14). Under hypoxic conditions, S-nitrosylation of the von Hippel- Lindau protein and HIF-alpha subunits prevents polyubiquitination and subsequent degradation of HIF (15), which may be substantial for the promotion of angiogenesis in tumor progression. Exogenous hypoxia changes the localization of prenylated proteins, including H-Ras. Under hypoxic conditions, H-Ras is translocated to the cytosol; conversely, hyperoxia increases the intracellular oxygen concentration and induces H-Ras translocation from the cytosol to the membranes. Translocation of Ras proteins depends on oxidative phosphorylation activity. Reductions in oxidative phosphorylation increase intracellular oxygen concentrations, which leads to the prenylation and membrane localization of proteins (16). Thus, the mobility and translocation of Ras depend on the oxidative status of cells. However, the distribution of S-nitrosylated Ras in the cytoplasm and plasma membranes under hypoxic conditions has not been described. The importance of relocated S-nitrosylated H-Ras (NO-H-Ras) is not completely understood in proliferation/ differentiation processes. In addition, Ras isoforms during intracellular translocation could change the oxidative metabolism either by direct actions with mitochondria or indirectly through modulation of mitochondria-associated endoplasmic reticulum (MAM). 

In this work, we used oxygen-sensitive undifferentiated (ND) and NGF-treated rat pheochromocytoma (PC12) cells to determine the levels of S-nitrosylation of soluble and membrane- bound NO-H-Ras under hypoxic and normoxic conditions. We found that hypoxia only decreased S-nitrosylation of soluble H-Ras in differentiated (D) PC12 cells and did not change the nitrosylation of membrane-bound H-Ras in D and ND PC12 cells. Our results showed that unmodified H-Ras enhanced the synthesis of ATP by mitochondria, whereas S-nitrosylation of H-Ras eliminated the stimulatory effects on ATP synthesis. 

## Materials and Methods

### PC12 cell line

**Fig.1:**
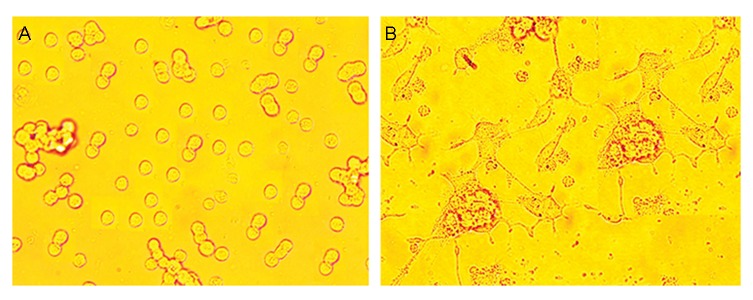
Cultured PC12 cells (Adh, ATCC® CRL1721.1™). A. Undifferentiated (ND) and B. Differentiated (D). pheochromocytoma (PC12) cells. ND PC12 cells were incubated in low serum-containing Dulbecco’s Modified Eagle Medium (DMEM) supplemented with nerve growth factor (NGF) 100 ng/ml for 5 days. The cells were examined 24 hours later, photographed, and scored for the presence of neurites. For each treatment, we counted 100 cells in each of 3 separate fields. Cells were scored positive if they contained one or more neurites >1 cell body diameter in length. The results presented are the mean ± SD of 10 independent experiments.

In our experimental study, we cultured PC12 (Adh; ATCC® CRL1721.1™) cells in T25 flasks (Greiner Bio-One GmbH, Cat. No.: 690 170) in a humidified atmosphere that contained 5% CO ^2^at 37˚C in high glucose Dulbecco’s Modified Eagle’s Medium (DMEM, ATCC® 30-2002™) supplemented with 10% heat- inactivated horse serum (Sigma-Aldrich), 5% fetal bovine serum (FBS, Sigma-Aldrich), 100 IU/ml penicillin and 50 µg/ml gentamycin sulphate. For the induction of differentiation, PC12 cells were incubated in low serum-DMEM that consisted of 1% heat-inactivated horse serum and 1% FBS, supplemented with nerve growth factor (NGF) 100 ng/ml for 5 days. The NGF-containing medium was replaced daily with fresh medium. We considered the cells to be D if one or more neurites were longer than the diameter of the cell body (Fig .1). Both D and ND cells were washed and plated at a density of 2×10^6^ on 100-mm-diameter dishes in serum-free DMEM. After a 24-hour period of starvation, cells were incubated with or without 100 µM sodium nitroprusside (SNP) and exposed to normoxia (N, 21% O_2_) or hypoxia (H, 1% O_2_) conditions for 6 hours. Hypoxic conditions (1% oxygen) were maintained by nitrogen gas in a Biospherix C-Chamber placed in a CO ^2incubatorandcontrolledbyacompact^oxygen controlling chamber (Proox, Model 110, BioSpherix, USA). 

### Preparation of membrane and soluble fractions from the PC12 cell line

After the 6-hour incubation, PC12 cells (3×10^6^ cells per sample) were removed from the cell culture flasks by scraping and pelleted by centrifugation at 300 x g. After centrifugation, we washed the PC12 cells twice with Ringer’s solution. Finally, the incubated PC12 cells were resuspended in subcellular fractionation buffer that consisted of 20 mM HEPES (pH=7.4), 10 mM KCl, 10 mM MgCl2, 1 mM EDTA, 1 mM ethylene glycol tetraacetic acid (EGTA), 250 mM sucrose, 1 mM dithiothreitol (DTT), and protease inhibitor cocktail (PI Cocktail III) (all reagents from Sigma-Aldrich, USA), then passed 10 times through a 25 guage needle using a 1 ml syringe. After lysis, the nuclei were sedimented at 720 x g for 5 minutes, and the post-nuclear fraction was centrifuged at 21000 x g for 30 minutes. The pellet was used as the membrane fraction and supernatants were used as the soluble fractions for the following analyses.

### Immunoprecipitation

Membrane fractions were resuspended in ice- cold solubilization buffer [20 mM Tris-HCl (pH=8.0), 137 mM NaCl, 10% glycerol, 1% Triton X-100, 2 mM EDTA] and incubated for 30 minutes at 4˚C. The unsolubilized material was removed by centrifugation (60 minutes at 20000 x g). Equal amounts of plasma membrane proteins and soluble proteins from each samples were incubated with the anti-H- Ras antibody (Abcam, UK) for 60 minutes at 4˚C, followed by the addition of protein A/G- agarose (20 μL per sample), then incubated overnight at 4˚C. After washing (12000 x g, 20 minutes), the protein A/G-agarose pellets were resuspended in 100 mM glycine, (pH=3.0) for 10 seconds. Next, a pretitrated volume of 1.0 M Tris (pH=9.5) was added to adjust the pH to 7.4. Immunoprecipitated H-Ras was used for the H-Ras S-nitrosylation detection experiments as well as for determination of total H-Ras. 

### Nitrosylated H-Ras detection

We used the S-nitrosylated Protein Detection Assay Kit (Cayman Chemical, Cat: 10006518) to detect nitrosylated H-Ras in the immunoprecipitated H-Ras preparation. Biotin-labeled NO-H- Ras was analyzed by the Western blot assay [immunolabeled by enhanced chemiluminescence (ECL) Streptavidin HPR, Amersham Biosciences, UK)] followed by visualization with ECL (Amersham Biosciences, UK) and analysis by densitometric scanning. Total H-Ras was detected after stripping the nitrocellulose membrane and incubation with the anti-H-Ras primary antibody (Abcam, dilution: 1:4000). Immunolabeled bands were visualized using ECL and analyzed by densitometric scanning. 

### Western blot

For analyzing the nitrosylated H-Ras and total H-Ras protein in the membrane and the soluble fraction, we boiled the samples at 90˚C for 5 minutes, after which they were separated by SDS- PAGE on 7.5-12% gradient gels, and transferred to nitrocellulose membranes. After blocking with 5% bovine serum albumin and 0.05% Tween 20 in Tris-HCl buffered saline, the membranes were incubated with the corresponding primary antibodies in the blocking solution. Immunolabeled bands were visualized using ECL and analyzed as described above. The band intensities were within the linear range of the labeled protein amount. Protein concentration was determined by a dye- binding method (Bio-Rad). 

### Nitrosylation of H-Ras

Functionally active H-Ras and H-Ras^V12^ were purchased from Jena Bioscience (Germany). We used the following procedure for nitrosylation of the Ras proteins (17). Briefly, equal volumes of 100 mM L-cysteine in 0.25 M HCl and 100 mM NaNO_2_ were mixed in dH_2_O and incubated for 10 minutes at room temperature in the dark. After incubation, the reaction mixture was diluted at a 4:1 ratio with buffer [20 mM Tris (pH=7.4)/1 mM diethylenetriamine pentaacetic acid]. The pH was adjusted to 7.4 with NaOH. S-nitroso-cysteine (0.1-1 mM) was added in 100-fold excess to H-Ras (25 μg) in the reaction buffer that contained 20 mM Tris (pH=7.4)/1 mM diethylenetriamine pentaacetic acid and incubated for 30 minutes at room temperature in the dark. After removal of excess low molecular weight compounds by Sephadex G-50 (Pharmacia) gel filtration, mannitol was added to an eluate fraction (final concentration: 0.1 M), and the protein solution (25 μg/ml) was separated in aliquots.

### Isolation of brain mitochondria

We used a discontinuous Percoll gradient to isolate non-synaptosomal mitochondria from the cortical bovine brain (18). Briefly, brain cortex (80 g) was removed and gently homogenized in a 10-fold volume of isolation buffer that consisted of 5 mM HEPES, 225 mM mannitol, 75 mM sucrose, and 1 mM EGTA at pH=7.5. Homogenate was centrifuged at 1250 x g for 5 minutes. The obtained supernatant was immediately centrifuged at 21000 x g for 10 minutes and the pellet was re-suspended in a cold 15% Percoll solution, then layered on 23 and 40% Percoll gradient solutions. The samples were centrifuged at 35000 x g for 8 minutes. The lower interphase was collected, washed twice with isolation buffer, and resuspended in isolation buffer without EGTA. This mitochondrial preparation does not contain Na,K-ATPase (plasma membrane marker) activity. The whole experiment was carried out under ice-cold conditions. All animal-related procedures were approved by the Laboratory Animal Care and Use Committee of I. Beritashvili Center of Experimental Biomedicine and conducted in accordance with the Guidelines of the European Communities Council, Directive 86/609/EEC. 

### Determination of mitochondrial H_2_O_2_ generation

Mitochondrial H_2_O_2_ generation was assessed by the scopoletin oxidation method. Freshly isolated mitochondria (100 μg of protein) were preincubated with 30 μg/ml digitonin (control) and 2.5 μg H-Ras (wild and mutant, nitrosylated and non-nitrosylated) for 5 minutes. Then, the suspension was incubated with 1 ml buffer that contained 10 mM HEPES, 5 mM MgCl_2_, 2 mM KH_2_PO_4_, 250 mM sucrose, 0.1% bovine serum albumin (BSA), 1 IU/ml horseradish peroxidase (HRP), and 100 nM scopoletin. We used 10 mM glutamate and 5 mM malate as substrates to activate the respiratory chain. Fluorescence was monitored at an excitation wavelength of 460 nm and an emission wavelength of 540 nm during 5 minutes. Calibration was performed by the addition of known quantities of H_2_O_2_.

### ATP production

We used the luminometric method to estimate ATP production. Briefly, 0.1 mg/ml of freshly isolated mitochondria were pre-incubated with 30 µg/ml digitonin (control) and 2.5 µg of H-Ras (wild and mutant, nitrosylated and non- nitrosylated) for 3 minutes, then added to the standard respiration buffer that consisted of 10 mM Tris-HCl (pH=7.4), 0.32 M mannitol, 8 mM inorganic phosphate, 4 mM MgCl ^2,0.08^mM EDTA, 1 mM EGTA, 0.1 mM Ap5A, and 0.2 mg/ml fatty acid-free BSA. We induced ATP production with the addition of 10 mM glutamate, 5 mM malate, 5 mM succinate, and 1 mM of ADP. After incubation at 25˚C for 10 minutes, the reaction was stopped by the addition of 0.6 M perchloric acid, then left on ice for 10 minutes, and subsequently centrifuged for 5 minutes at 15300 x g. The supernatant was collected and neutralized with 1 M KOH. ATP was quantified by the luciferin/luciferase assay (Sigma). 

### Statistical analysis

Statistical analyses were performed by either an unpaired t test or one-way ANOVA, and Scheffe’s post hoc analysis where appropriate. Results were considered significant at P<0.05. The results were expressed as the mean ± SEM of the groups from at least three independent experiments. 

## Results

We tested the effect of hypoxia on H-Ras nitrosylation. Initially, we exposed D and ND PC12 cells to normoxic and hypoxic conditions. 

**Fig.2:**
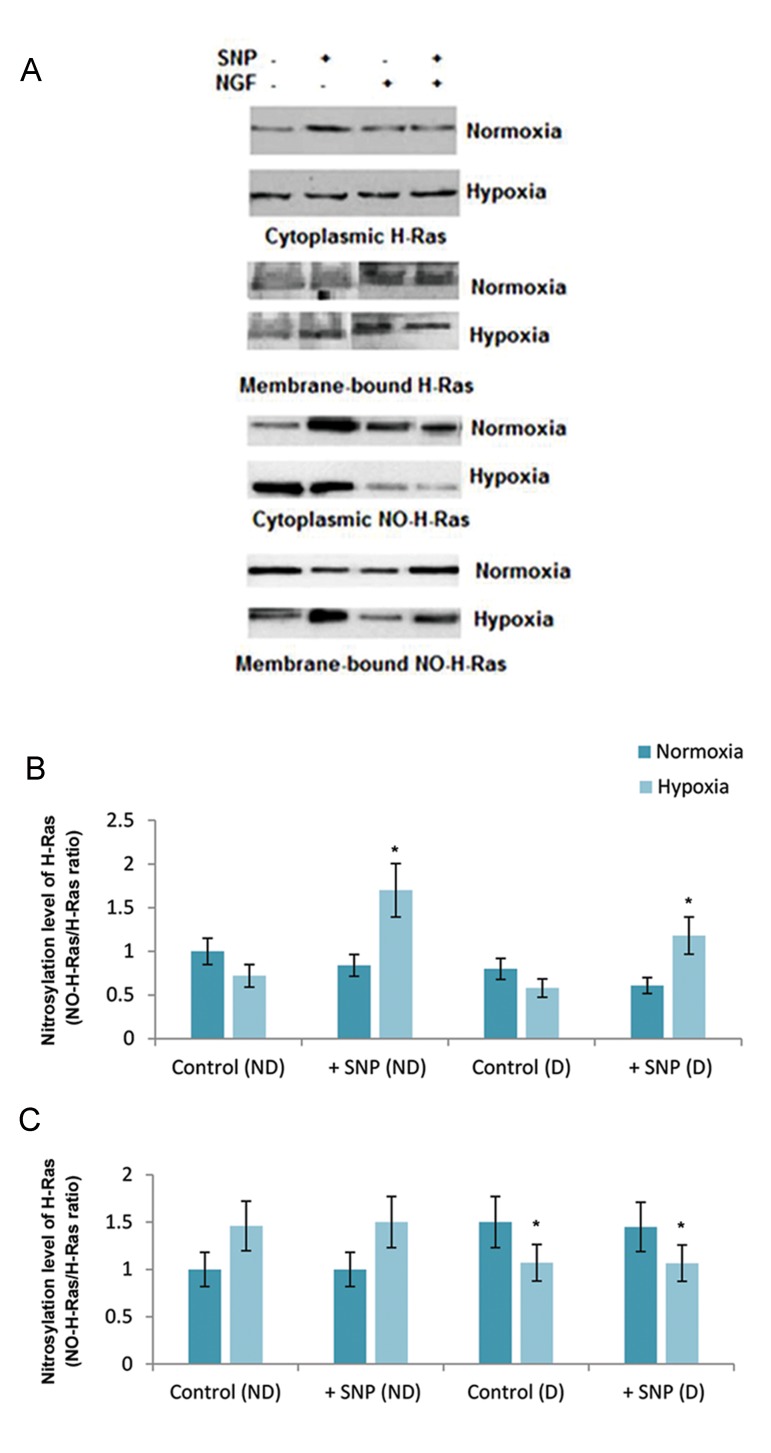
Analysis of total soluble, membrane-bound H-Ras and soluble, membrane-bound nitrosylated-H-Ras (NO-H-Ras), in differentiated (D) and undifferentiated (ND) pheochromocytoma (PC12) cells under normoxic and hypoxic conditions. A. Western blot analysis of total soluble and membrane-bound H-Ras, as well as soluble and membrane-bound nitrosylate-H-Ras (NO-H-Ras), in D and ND pheochromocytoma (PC12) cells under normoxic and hypoxic conditions. For the immunoblots, 50 μg of total proteins from each fraction were loaded into each well, resolved by sodium dodecyl sulfate polyacrylamide gel electrophoresis (SDS-PAGE), transferred to nitrocellulose membranes, and probed with anti-H-Ras and biotin-streptavidin HPC for NO-H- Ras. The blot is representative of three similar experiments. We normalized NO-H-Ras levels to total H-Ras for, B. Membrane- bound H-Ras, and C. Soluble-H-Ras. Significance level was set at *P<0.05 and compared with the control group.

Next, we determined the total H-Ras content by Western blot analysis of the membrane and soluble proteins. We observed that hypoxia, per se, increased the expression of soluble H-Ras and did not change the amount of membrane-bound H-Ras in ND cells. However, in NGF-treated D cells, hypoxia enhanced the expression of soluble and membrane-bound H-Ras (Fig .2A). 

Of note, the addition of SNP to the incubation media of ND cells significantly raised the expression of soluble H-Ras during normoxia. 

In ND cells, the content of membrane-bound H-Ras increased in the presence of SNP and decreased in NGF-treated cells under the hypoxic condition. 

Next, we determined the levels of membrane- bound and soluble NO-H-Ras under normoxic and hypoxic conditions in both cell types. 

We found that hypoxia differently affected S-nitrosylation of soluble H-Ras, as well as membrane-bound H-Ras in ND and D cells (Fig .2A). After adjusting densimetric data to the amount of total H-Ras, we observed that endogenous nitrosylation of membrane-bound H-Ras did not change during hypoxia, in ND or D cells (Fig .2B,C). However, the addition of NO donor-SNP to the ND and D cells greatly increased nitrosylation of H-Ras under hypoxic conditions. Hypoxia reduced the nitrosylation of soluble H-Ras in D cells both in the presence and absence of SNP but did not affect the nitrosylation of H-Ras in ND cells. Under normoxic conditions, the levels of nitrosylation did not change in any of the cell types

**Fig.3:**
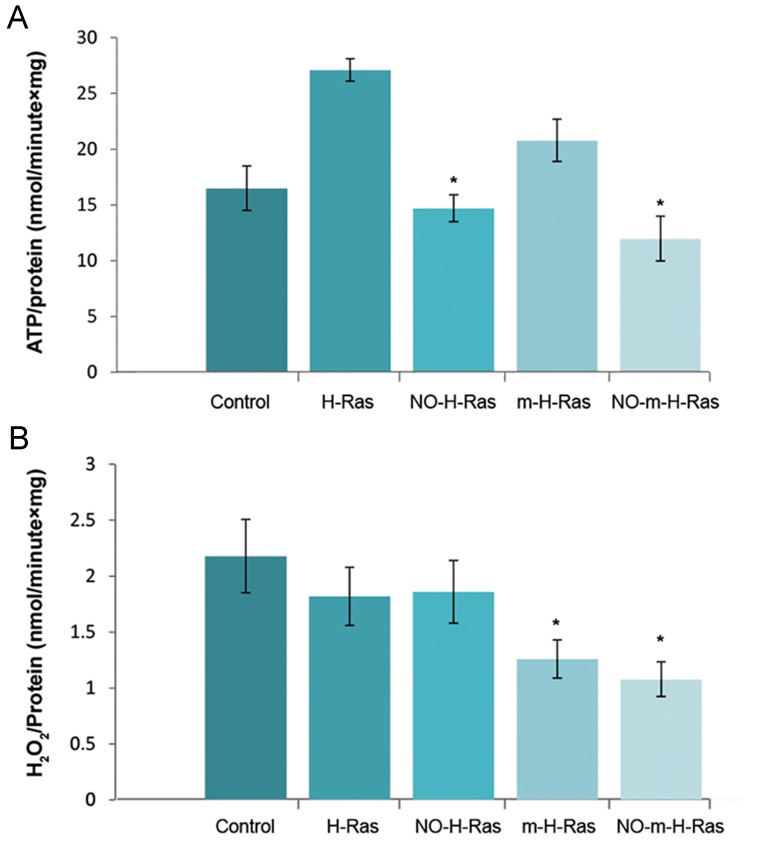
The action of normal H-Ras or mutated H-RasV12 (m-HRas) and normal nitrosylated H-Ras (NO-H-Ras) or mutated nitrosylated H-Ras^V12^(m-NO-H-Ras) on oxidative metabolism of mitochondria. A. Changes in ATP synthesis and B. H_2_O_2_ generation in brain mitochondria under the action of H-Ras, NO- H-Ras and m-H-Ras, NO-m-H-Ras. Freshly isolated mitochondria were incubated either with H-Ras and NO-H-Ras, or m-H-Ras and NO-m-H-Ras. ATP production and H_2_O_2_ generation were determined. Data represented are mean ± SEM of results from four separate experiments performed in duplicate. *; P<0.05 was compared by the t test with the corresponding control.

Normal H-Ras, as well as mutant H-Ras ^V12^(m-H-Ras) could interact with the intracellular structures, including mitochondria that change A B C the oxygen consumption and production of ROS (19). In order to investigate the action of nitrosylated H-Ras on oxidative metabolism, we have treated the mitochondria with H-Ras and H-RasH-RasV12 to determine ATP-synthesis and H_2_O_2_-production(Fig .3A,B). We found that treatment of mitochondria by H-Ras or H-Ras ^V12^ caused an increase in ATP synthesis, while the stimulatory effects of both types of Ras were eliminated after nitrosylation of proteins. Interestingly, neither unmodified nor nitrosylated H-Ras changed the mitochondrial production of H_2_O_2_. However, the formation of peroxides significantly decreased after treatment of mitochondria by m-H- Ras ^V12.Inthiscase,nitrosylationofH-Rasdid^not affect the production of ROS. These data suggested that H-Ras increased the synthesis of mitochondrial ATP only in an unmodified state and protein nitrosylation eliminated the stimulatory effect of H-Ras. 

## Discussion

NO plays a major role in tissue function during hypoxia (15). A major mechanism by which NO regulates the hypoxia signaling pathway, as well as numerous other cellular targets, is S-nitrosylation of proteins (11). In addition to the wide range of regulatory proteins, NO has been shown to react with Ras and other Ras- related GTPases (4). Under hypoxic conditions, H-Ras is activated by S-nitrosylation (20) which initiates translocation of H-Ras in the cytosol (16). 

NO-modifications of Ras in different subcellular compartments regulate different downstream signaling pathways, which lead to various outcomes in proliferation, differentiation or apoptosis (6). The trafficking and relocalization of H-Ras are regulated by the palmitoylation/depalmitoylation cycles of terminal Cys-Cys181 and Cys184. These Cys are potential sites of S-nitrosylation and can be modified with high concentrations of NO (7). The reactivity of NO may be increased by hypoxia, which possibly affects the processes of terminal Cys nitrosylation. 

Here, we analyzed the levels of NO-H-Ras in hypoxic and normoxic conditions in ND and NGF-treated PC12 cells with and without treatment of cells with SNP. The following conclusions could be proposed from our data: there were no significant differences observed in nitrosylation of soluble or membrane- bound H-Ras between D and ND PC12 cells in normoxic conditions; hypoxia decreased nitrosylation of the soluble form of H-Ras but did not affect nitrosylation of membrane- bound H-Ras. We found that under hypoxic conditions, nitrosylation of soluble H-Ras reduced both in the presence and absence of the SNP, which suggested that the NO concentration did not limit nitrosylation of soluble H-Ras in hypoxia. Differentiation of PC12, in this case, did not affect nitrosylation of H-Ras. These data supported a previous investigation which observed that NO was unable to activate the H-Ras-dependent signaling pathway in PC12 cells during differentiation (21). Our results showed that under hypoxic conditions, endogenous nitrosylation of membrane-bound H-Ras was unaffected; however, nitrosylation of H-Ras greatly increased in the presence of SNP. These data suggested that a high concentration of NO could alter the nitrosylation of membrane- bound H-Ras. 

Apparently, the difference in sensitivity to SNP between membrane-bound and soluble H-Ras in our experiments was due to various basal nitric oxide synthase (NOS) activities in the ND and NGF-treated cells. Subcellular localization of NOS in PC12 varies with the cellular activity. Extracellular signals in PC12 cells induce the translocation of NOS from the cytoplasm to plasma membranes where it can be incorporated in the ternary complex of the NMDA receptor (22). Membrane-bound H-Ras is located in proximity to neuronal NOS (nNOS). Short-range S-nitrosylation signaling to H-Ras occurs in this case (23). Preferential localization of the non-inducible isoform of NOS at the plasma membrane produces higher levels of NO. Hence, S-nitrosylation of proteins in this region is more efficient than in the endomembrane compartments. Therefore, Cys residues of H-Ras may be occupied as a result of NOS activity. Different intracellular localization and activities of NOS in PC12 cells may explain various amounts of H-Ras sensitivity to SNP. 

Mitochondria play a critical role in oxygen sensing during hypoxia by releasing ROS to the cytosol (24). Treatment of cells by NGF induces association of mitochondria with the plasma membrane and microtubular cytoskeleton (25). In this complex, Ras initiate the formation of H_2_O_2_ by activating superoxide dismutase, maintaining long term ERK1/2 activation, and differentiation of PC12 cells. Besides peroxide production, Ras may directly change mitochondrial metabolism through modification of the MAM proteins (26). MAM contains Bcl- 2 family proteins that dynamically interact with IP3R to coordinate mitochondrial Ca^2+^ transfer and alter cellular metabolism to increase the cells’ bioenergetic capacities, particularly during stress (27). A direct association of Ras with Bcl-2 (28) or Bcl-xL (29) has been demonstrated. All three Ras proteins, K-, Nand H-Ras, interact with Bcl-2; however, their mitochondrial localization is differentially regulated by various environmental stimuli (30). Of note, H-RasV12 significantly increases mitochondrial metabolism compared to H-Ras (18). Our results have agreed with these observations since H-Ras, as well as H-RasV12, have enhanced the production of mitochondrial ATP. However, both forms of nitrosylated H-Ras are unable to stimulate ATP synthesis, which suggests that S-nitrosylation changes the interaction of H-Ras with mitochondrial targets. On the other hand, mutated H-RasV12 significantly decreases ROS formation and nitrosylation. In this case, it does not eliminate the inhibitory effect of H-Ras. In conclusion, our results have suggested that hypoxia decreases the nitrosylation of soluble H-Ras which, in turn, may underlie in the elevation of oxidative metabolism and mitochondrial ATP-synthesis.

## Conclusion

Our recent findings propose that hypoxia can decrease S-nitrosylation of soluble H-Ras in D PC12 cells and abolish the inhibitory effect of nitrosylated H-Ras in mitochondrial oxidative metabolism. 
